# Biological effects of selective COX-2 inhibitor NS398 on human glioblastoma cell lines

**DOI:** 10.1186/s12935-020-01250-7

**Published:** 2020-05-13

**Authors:** Paola Palumbo, Francesca Lombardi, Francesca Rosaria Augello, Ilaria Giusti, Vincenza Dolo, Pietro Leocata, Maria Grazia Cifone, Benedetta Cinque

**Affiliations:** Department of Life, Health & Environmental Sciences, University of L’Aquila, 67100 L’Aquila, Italy

**Keywords:** Glioblastoma, U87MG, T98G, Glioma stem cells, Inflammation, COX-2, COX-2 inhibitor, NS398, Autophagy, Extracellular vesicles

## Abstract

**Background:**

Cyclooxygenase-2 (COX-2), an inflammation-associated enzyme, has been implicated in tumorigenesis and progression of glioblastoma (GBM). The poor survival of GBM was mainly associated with the presence of glioma stem cells (GSC) and the markedly inflammatory microenvironment. To further explore the involvement of COX-2 in glioma biology, the effects of NS398, a selective COX-2 inhibitor, were evaluated on GSC derived from COX-2 expressing GBM cell lines, i.e., U87MG and T98G, in terms of neurospheres’ growth, autophagy, and extracellular vesicle (EV) release.

**Methods:**

Neurospheres’ growth and morphology were evaluated by optical and scanning electron microscopy. Autophagy was measured by staining acidic vesicular organelles. Extracellular vesicles (EV), released from neurospheres, were analyzed by transmission electron microscopy. The autophagic proteins Beclin-1 and LC3B, as well as the EV markers CD63 and CD81, were analyzed by western blotting. The scratch assay test was used to evaluate the NS398 influence on GBM cell migration.

**Results:**

Both cell lines were strongly influenced by NS398 exposure, as showed by morphological changes, reduced growth rate, and appearance of autophagy. Furthermore, the inhibitor led to a functional change of EV released by neurospheres. Indeed, EV secreted by NS398-treated GSC, but not those from control cells, were able to significantly inhibit adherent U87MG and T98G cell migration and induced autophagy in recipient cells, thus leading to effects quite similar to those directly caused by NS398 in the same cells.

**Conclusion:**

Despite the intrinsic diversity and individual genetic features of U87MG and T98G, comparable effects were exerted by the COX-2 inhibitor NS398 on both GBM cell lines. Overall, our findings support the crucial role of the inflammatory-associated COX-2/PGE2 system in glioma and glioma stem cell biology.

## Background

Gliomas are classified by the World Health Organization (WHO) grade criteria (I to IV) into multiple specific histologic subtypes, based on cell type of origin and molecular characteristics [1]. Grade IV Glioblastoma (GBM) is one of the most lethal brain cancers worldwide since it is the most highly infiltrating and aggressive tumor of the central nervous system, characterized by a high degree of genetic and cellular heterogeneity. Nowadays, for the treatment of GBM, surgical resection is usually followed by radiotherapy or radiotherapy plus chemotherapy with temozolomide (TMZ) [2]. These therapeutic approaches have increased median patient survival of 15-23 months [3, 4], but GBM often relapses due to the presence of tumor-initiating cells, called GBM stem cells (GSC), promoting tumor recurrence, therapy resistance and metastasis. GBM is characterized by the presence of an inflammatory milieu, which is emerging as a target for new treatment modalities [5–7].

In our previous studies, the role of NOS2 (Nitric Oxide Synthase 2), a key inflammatory marker highly expressed in GBM, has been evaluated in GBM cell lines and primary cultures, pointing out the correlation between NOS2 expression and ability to generate neurospheres [8]. We also reported that selective NOS2 inhibition by 1400W (*N*-(3-(Aminomethyl)benzyl)acetamidine) was able to reduce proliferation, migration, colony formation, and neurospheres’ generation ability of U87MG and T98G cell lines [9].

Cyclooxygenase-2 (COX-2), another pivotal inflammatory marker, is an inducible enzyme that catalyzes the first step of the synthesis of prostanoids by converting arachidonic acid into prostaglandin PGH2 and prostaglandin E2 synthase delivers PGE2 [10]. Under normal conditions, COX-2 expression is low or absent at basal levels but is rapidly induced by several stimuli, and, unlike the constitutive isoform COX-1, COX-2 is frequently overexpressed in cancerous tissues, including glioma and is implicated in cell proliferation, migration, metastatic process, angiogenesis, as well as in cancer stem-like phenotype promotion [11, 12]. The enhanced expression of COX-2 protein has been correlated with many aggressive aspects of the disease, such as rate of GBM cell proliferation [13], histopathological grade of glioma [14, 15], and poor prognosis and survival [16–19]. Furthermore, evidence has been reported that the COX-2 pathway, through the stimulation of the GSC self-renewal and proliferation, could contribute to chemo- and radio-resistance [20–22]. Hence, COX-2 has been proposed as a strong predictor of poor survival and aggressiveness [12, 18] as well as its targeting with specific COX-2 inhibitors is emerging as a potential anti-glioma strategy [12, 14, 23, 24]. Indeed, COX-2 overexpression was found to correlate with GBM cell migration and invasion as well as increased vascular endothelial growth factor (VEGF) levels and high microvessel density [25–28]. The selective COX-2 inhibitor rofecoxib, in combination with low dose of TMZ, has been proposed as an antiangiogenic strategy to treat GBM [29]. The GBM-associated hypoxic microenvironment has been reported to induce COX-2 expression [30] and COX-2 and its product PGE2 are well known immunosuppressive factors in various cancers, including GBM [31]. Taking into account that chemotherapy and radiotherapy can induce COX-2 activity in GBM cells with PGE2 synthesis which in turn causes overexpression of immunosuppressive cytokines and inhibits T cell infiltration and proliferation, several findings suggest that the conventional treatment-induced immunosuppression can be counteracted by NSAIDs such as diclofenac, NS398, celecoxib [32–34].

NS398, a sulphonamide derivative, is a specific COX-2 inhibitor with an IC50 of 3.8 × 10^−6^ M on the enzyme isolated from sheep placenta [35], without affecting COX-1 activity at concentrations exceeding 100 mM (> 50-fold potency for COX-2 over COX-1) [36]. The anti-proliferative effect of NS398 on GBM cell lines (i.e., U87MG and U251MG) and relative neurospheres was firstly reported by Joki et al. [14]. Of note, a significant reduction in glioma growth was registered in mice bearing tumor xenografts after 2 weeks of treatment with NS398 able to reduce PGE2 secretion from tumor xenografts [37, 38].

Taking into account previous reports which showed that anti-glioma effects of some clinical and experimental approaches are associated with upregulation of the autophagic pathway [39, 40], it would be of interest to verify if the COX-2 inhibition can also exert the same action. Autophagy is a highly dynamic and conserved-catabolic process responsible for degrading damaged cellular structures and proteins by lysosomal digestion and recycling macromolecules. It occurs at low basal levels in all cells to perform homeostatic functions and is rapidly induced during different forms of metabolic stress [41–44]. Recently, in vitro and in vivo studies on glioma models have reported an increase of autophagy levels in response to different antitumor approaches [39, 45–47]. TMZ has been reported to influence cell viability by modulating autophagy [48]. The activation of this pathway is crucial for the susceptibility to the treatment [49, 50]. Of interest, it been recently demonstrated that chaperone mediated autophagy (CMA) is the main mechanism by which TMZ treatment decreases hypoxia-inducible factor-1α (HIF-1α) activity in sensitive cells, thus improving responsiveness by promoting cell apoptosis. Moreover, the relation between mitochondrial reactive oxygen species (ROS), CMA induction, and TMZ-sensitiveness in GBM has been showed [51].

A strict interplay between autophagy and EV release, with important implications for normal physiology and disease states has been well recognized and documented [52, 53]. The significance of EV signalling in cancer, and the role of autophagy in multiple phases of tumorigenesis has been reviewed [54]. However, the importance of the crosstalk between EV and autophagy in cancer, including GBM, is not fully clarified [53].

**Fig. 1:**
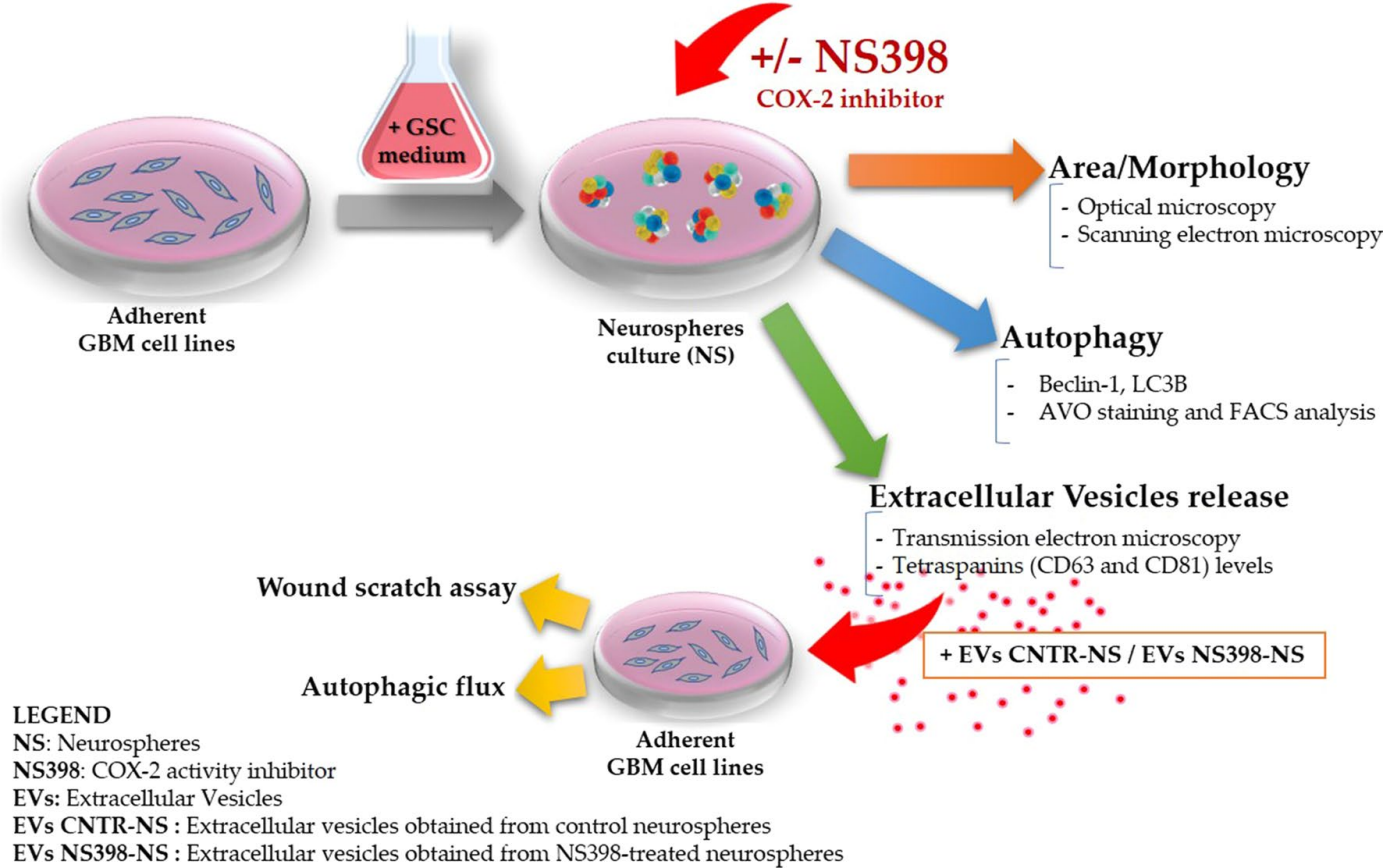
Scheme of experimental design

Based on the above evidence, the purpose of the present study was to investigate the ability of the COX-2 selective inhibitor, NS398, to influence the biology of neurospheres derived from U87MG and T98G cells, which have been chosen being considered an appropriate model of heterogeneous GBM. The U87MG human GBM cell line, showing an epithelial morphology, expresses the isocitrate dehydrogenase (IDH)-wild type, O6-methylguanine-DNA methyltransferase (MGMT) promoter methylated, MGMT-negative, and a wild-type p53 [55–57]. The T98G human GBM cell line, showing a polymorphic with fibroblast-like, polygonal morphology, expresses the isocitrate dehydrogenase (IDH)-wild type and MGMT and also express p53 mutated [55, 58, 59]. Cell growth, autophagy flux, and EV secretion were evaluated after treatment with COX-2 inhibitor. The influence of NS398 was also assessed on migration rate and autophagy in adherent U87MG and T98G. In addition, the effect of EV released by neurospheres previously incubated with or without NS398 was evaluated on the adherent GBM cell lines (recipient cells), according to the scheme shown in Fig. 1.

## Methods

### GBM cell coltures, neurosphere generation and treatments

Human GBM grade IV cell lines, U87MG and T98G were purchased from the European Collection of Authenticated Cell Cultures (ECACC) and American Type Culture Collection (ATCC), respectively. Both GBM cell lines were grown as adherent cells in DMEM (Dulbecco’s Modified Eagle Medium) supplemented with 10% (v/v) of fetal calf serum (FCS), 2 mM l-glutamine, 100 U/mL penicillin, and 100 mg/mL streptomycin (standard medium, St-M) (Euro Clone, West York, UK). The cells were maintained in a humidified incubator with 5% CO2 at 37 °C. Subcultures were performed every 3 days; after reaching 70/80% confluence adherent cells were detached using trypsin–EDTA solution (Euro Clone, West York, UK). The culture medium was totally replaced by centrifugation for 10 min at 400*xg*. Cell viability was determined using trypan blue solution (0.04% final concentration in PBS, Euro Clone, West York, UK), and neurospheres were visualized by optical microscopy (Eclipse 50i, Nikon Corporation, Japan). Here, to perform most of the experiments, we chose the neurosphere model since sphere formation is a typical characteristic of cancer stem cells when cultured in serum-free medium due to their self-renewal ability [60]. In addition, most importantly, GBM neurospheres are able to give rise to a tumor in xenograft model [61]. As previously described [8], for neurosphere (NS) generation, 5x10^5^ cells were seeded onto T25 cell culture flasks and cultured in suspension using a serum-free medium DMEM/F12 (1:1, vol/vol) supplemented with B27 reagent (Life Technology Corporation, CA, USA), EGF (20 ng/mL), and FGF-2 (20 ng/mL) (both purchased from ImmunoTools, 26169, Friesoythe, Germany), penicillin/streptomycin and glutamine. Neurosphere media were replaced every 3 days. All reagents for cell biology and consumables were purchased from EuroClone (EuroClone, West York, UK). The neurosphere cultures were routinely evaluated by flow cytometry and compared to adherent GBM cells for phenotypic marker expression. For cytofluorimetric analysis, we used a FACSCalibur supplied with CellQuest software (Becton–Dickinson, San Jose, CA). The primary antibodies were used for immunophenotypic analysis: anti-β-tubulin III (Alexa Fluor 488, Catalog No. 560338), anti-SOX-2 (PE Mouse, Catalog No. 560291), anti-nestin (Alexa Fluor 647, Catalog No. 560393), all acquired from Becton–Dickinson (BD), anti-GFAP (Glial fibrillary acidic protein; Catalog No. G9269 Sigma–Aldrich). Secondary antibody anti-rabbit FITC conjugated (Millipore) was used for detection of GFAP positive cells. Neurospheres were treated in the absence or presence of the selective COX-2 inhibitor, NS398 (N-[2-(Cyclohexyloxy)-4-nitrophenyl]methanesulfonamide) acquired from Sigma Aldrich (Sigma Aldrich, Saint Louis, MO, USA). Cells were firstly treated with different concentrations of NS398, as follows: 10 µM, 100 µM, and 200 µM for 48 h. According to manufacturer instruction, the COX-2 inhibitor was stored as stock solutions in DMSO at − 20 °C and diluted in cell culture medium just before use. The treatment with the vehicle alone was referred to as “control”, while the term “not-treated” was used for cells incubated with only culture medium. For all the parameters evaluated in this work, DMSO, used to dissolve NS398 at 0.6% (v/v) final concentration, did not have any effects on its own. Morphology and size of neurospheres were visualized by microscope Nikon Eclipse TS100 at the initial time (T0) and after 48 and 72 h from addition of COX-2 inhibitor NS398 and at a longer incubation (range 96–144 h), after subculturing, with fresh medium and without inhibitor. To determine the area of the neurospheres, 10 bright field images (at 4× magnification) were randomly taken at each time points under the inverted microscope (Nikon Eclipse TS100). The size of the neurospheres was determined using Image J software. The neurospheres’ average area (total area/number of neurospheres) was expressed in mm^2^, as previously reported [9]. After NS398 exposure, in order to assess cell viability, U87MG- and T98G-derived neurospheres were enzymatically dissociated with the Accutase™ solution (from PAA-GE Healthcare Life Sciences Bio-Sciences AB, SE-751 84 Uppsala Sweden) for about 10 min at 37 °C and stained with trypan blue dye exclusion assay recording live and dead cells number. Cell viability was evaluated by flow cytometric analysis by propidium iodide (PI) staining. PI staining is commonly used for identifying dead cells in a population and, since it can penetrate only in cells with disrupted membranes, is excluded from viable cells. PI staining was measured with FACS analysis. Cells were incubated with PI for 30 min and analyzed by flow cytometer (BD Instruments Inc., San José, CA, USA), equipped with BD CellQuest Software (BD Instruments Inc.).

### Prostaglandin 2 (PGE2) level assay

The levels of secreted PGE2 in the cell supernatants were quantified using an prostaglandin E2 enzyme-linked immunosorbent assay (ELISA) kit (Cayman Chemical Company, Ann Arbor, MI, USA) as described in the manufacturer’s instructions and presented as pg/mL. Breafly, neurospheres were treated in presence or absence of NS398 (10 µM, 100 µM, and 200 µM) for 48 h; cell culture supernatants were assayed at a final dilution of 1:10 for U87MG and not diluited for T98G cultures. The least detectable PGE2 concentration was 7.8 pg/mL.

### Western blot analysis

Treated or not neurosphere cell pellets were homogenized in ice-cold RIPA buffer (phosphate buffer saline pH 7.4) supplemented with 0.5% sodium deoxycholate, 1% NP40, 0.1% SDS, 5 mM of EDTA (ethylenediaminetetraacetic acid) and 100 mM of protease inhibitor cocktail (Sigma Aldrich, Saint Louis, MO, USA). Protein lysates (25 μg/lane for COX-2, β-actin, LC3B and Beclin-1 and 10 μg/lane for EV proteins, respectively) were separated on 10% SDS–polyacrilamide gel under reducing conditions with β-mercaptoethanol 5% and electroblotted on to nitrocellulose membranes. For CD63 and CD81 detection, equal amount of EV proteins (10 μg/lane) were resolved by 10% SDS polyacrylamide gels under reducing conditions with β-mercaptoethanol 5% according to standard procedures, and proteins were transferred onto nitrocellulose membranes. Nonspecific binding sites were blocked by 5% non-fat dry milk in Tris buffered saline for 1 h at room temperature. Afterward, membranes were incubated overnight at 4 °C with the following primary antibodies: rabbit monoclonal anti-COX-2 (Cell Signaling, Danvers, Massachusetts, United States), mouse monoclonal anti-β-actin antibody (Santa Cruz Biotechnology, Santa Cruz, CA), rabbit polyclonal anti-LC3B (Thermo Fisher, Waltham, Massachusetts, United States), mouse monoclonal anti-Beclin-1 (Origene, 9620 Medical Center Drive Suite 200 Rockville, MD, USA), mouse monoclonal anti-CD63 and mouse monoclonal anti-CD81 were acquired from Novus Biological (Briarwood Avenue, Centennial, USA). As secondary antibodies, peroxidase conjugated anti-rabbit and anti-mouse IgG 1:2000 (Millipore EMD, Darmstadt, Germany) were used. Immunoreactive bands were visualized by enhanced chemiluminescence (ECL, Amersham Pharmacia Biotech), according to the manufacturer’s instructions. Band relative densities were determined using a chemiluminescence documentation system ALLIANCE (UVITEC, Cambridge UK), and normalized to relative β-actin band, using software provided by the company.

### Scanning electron microscopy

Scanning electron microscopy (SEM) was carried out on U87MG- and T98G-derived neurospheres, that were previously untreated or treated with NS398 (100 µM for 48 h) and then left to adhere overnight on coverslips that were pre-coated with poly-lysine (30 µg/ml) (Sigma Aldrich, Saint Louis, MO, USA). Neurospheres were fixed with 2% glutaraldehyde (Electron Microscopy Sciences, Hatfield, PA, USA) in PBS for 30 min and the coverslips briefly rinsed with PBS and water; afterward they were dehydrated in ethanol solutions 30–50–70–90% in H_2_O and three times 100%, for 5 min each. For HMDS drying, the samples were immersed in HMDS:EtOH 1:2, HMDS:EtOH 2:1 and HMDS 100% for 3 min each. (Electron Microscopy Sciences, Hatfield, PA, USA) and then the excess HMDS was blotted away by filter paper. The samples were then transferred to a desiccator for 25 min. to avoid water contamination, mounted on stubs, sputter-coated with chromium in a Quorumtech Q 150T ES Turbo chromium sputter, and detected via a Zeiss Gemini SEM 500.

### Autophagy detection: fluorescent staining of acidic vesicular organelles (AVO)

For autophagy evaluation the acridine orange (AO) staining was performed to detect the presence of acidic vesicular organelles (AVOs), an hallmark of autophagy process. Before staining with AO, adherent GBM cell lines (1 × 10^5^) were previously plated onto glass round coverslips in 12-well plates, allowed to attach by overnight incubation, and then exposed to NS398 (100 μM for 48 h). Before staining with AO, neurospheres from both U87MG and T98G were disaggregated with Accutase solution and plated onto glass round coverslips coated with poly-lysine for 12 h and then treated with NS398 similarly to adherent cells. AO (Sigma Aldrich, Saint Louis, MO, USA) was added at a final concentration of 0.01 μg/mL in PBS at room temperature for 10 min and in presence of RNase A (6 μg/10^6^ cell, Sigma Aldrich, Saint Louis, MO, USA) both in U87MG and T98G adherent and neurophere cultures. Cells were washed twice with PBS, and examined under a fluorescence microscope. AO accumulates in acidic organelles in a pH-dependent manner in cells. At neutral pH, it emits green fluorescence, but within acidic environments, AO becomes protonated and gets trapped within the organelle and then aggregates and emits red fluorescence. In AO-stained cells, the cytoplasm and nucleolus fluoresce bright green, whereas acidic compartments fluoresce bright orange/red. The intensity of the orange/red fluorescence is proportional to the degree of acidity and/or the volume of the cellular acidic compartment. To detect and quantify AVOs (autophagy cells), FACS analysis was performed by FACSCalibur flow cytometry (BD Instruments Inc., San José, CA, USA) equipped by Cell Quest software (BD Instruments Inc.). Cells were analyzed using the 488-nm excitation detector (green fluorescence/FL1) and the 540-nm emission detector (red fluorescence/FL3). U87MG and T98G cells subjected to serum withdrawal for at least 4 h have been used as positive control of autophagy.

### Isolation and characterization of neurospheres-derived extracellular vesicles

The EV were isolated from U87MG- and T98G-derived neurospheres cell media by ultracentrifugation, as previously described [62]. Briefly, all of the supernatants from neurospheres cultures treated in presence or absence of NS398 for 48 h were collected in aseptic working conditions and initially centrifuged in clear and sterile tubes at 4 °C at 600×*g* for 10 min and 1500*xg* for 30 min to remove cellular debris. The resulting supernatants were centrifuged at 100,000×*g* (Rotor 70Ti, Quick-Seal Ultra-Clear tubes, k_adj_ 221, brake 9) for 2 h at 4 °C in an Optima XPN-110 Ultracentrifuge (Beckman Coulter, Brea, CA, USA). The pelleted EV were resuspended in PBS. The quantity of EV was double measured by determining the total protein concentration in the preparations using the BCA Protein Assay Kit (Pierce, Rockford, IL, USA), following the manufacturer’s instructions. The samples were immediately used or stored at − 20 °C. Identification of purified EV was achieved by morphological examination by transmission electron microscope.

### Transmission electron microscopy

To further characterize the EV obtained from GBM neurospheres and to confirm their ultrastructural morphology, transmission electron microscopy (TEM) was performed on EV. After collection, EV were resuspended and diluited in PBS and, according to proper dilutions, the samples were adsorbed onto 300-mesh carbon-coated copper grids (Electron Microscopy Sciences) for 5 min in a humidified chamber at room temperature. EV on grids were fixed in 2% glutaraldehyde (Electron Microscopy Sciences) in PBS for 10 min and then briefly rinsed in Milli-Qwater. Grids with adhered EV were examined with a Zeiss Gemini SEM 500 equipped with a STEM detector at 20 kV and at a 3.0 mm working distance, after negative staining with 2% phosphotungstic acid, brought to pH 7.0 with NaOH [62].

### Extracellular vesicles labeling

Fluorescent staining of EV is a commonly used method to verify their uptake in target cell evaluating the in vitro and in vivo distribution. EV were stained in aseptic working conditions, with a PKH26 Red Fluorescent Cell Linker kit (Sigma-Aldrich, Saint Louis, MO, USA) according to according to the manufacturer’s protocol. Briefly, EV pellets were resuspended in 1 mL Diluent C. To each samples 6 μL PKH26, a lipophilic fluorescent dye, were added using a laminar flow biosafety hood. The exosome suspension was mixed for 30 s with the stain solution and incubated for 5 min at room temperature. The labeling reaction was stopped by adding 2 ml of 1% BSA in sterile PBS. Labeled EV were ultracentrifuged as previously described. A negative technical control with same volume of diluent C and PKH2 as samples was also ultracentrifuged to check if the free dye does not precipitate. Afterward, U87MG and T98G cells were incubated for 18 h at 37 °C in a 95% air 5% CO_2_ atmosphere, with PKH26-labeled EV (30 µg) from respective neurospheres previously treated with NS398. The coverslips were mounted with Vectashield^®^ Antifade Mounting Medium with DAPI (Vector Laboratories, Inc., Burlingame, CA, USA), and the EV internalization was viewed under a fluorescent microscopy (Nikon, Eclipse 50i, Tokyo, Japan) and the images were acquired at 100× magnification.

### Scratch wound assays

Wound-healing assay was used to detect the migration ability of GBM cell lines U87MG and T98G following NS398 exposure. Adherent U87MG and T98G cells were cultured in standard conditions at 6 × 10^4^/cm^2^ in multiwell plates, to permit the monolayer formation, until treatment with NS398 (100 μM for 48 h) and wounded with a 200 µl pipet tip. Culture medium was immediately removed (along with any suspended cells), the scratched monolayers were washed with PBS and cultured with a fresh serum supplemented culture medium (10% FCS) at 37 °C in a 5% CO_2_ humidified atmosphere in the absence or presence of 100 µM NS398. The wound-closing process was observed every hours and phase contrast photographs at 8 and 24 h were taken. The experiments were conducted in duplicate, and at leasts three fields for well were captured. The TScratch software was used to measure percentage of wound closure; the software automatically calculates the portion of area occupied by the cells by a mathematical model. The quantification of a relative scratched monolayer closure (wound closure) was performed according to the equation where Tn is a specific time point after the scratching:

$$\% {\text{ Relative wound closure }} = \frac{{\left[ {\% {\text{ of scratched area at T}}0 - \% {\text{ of scratched area at Tn}}} \right]\left( { \times 100} \right)}}{{\left[ {\% {\text{ of scratched area at T}}0} \right]}}$$

### Statistical analysis

Statistical analysis was performed by GraphPad Prism 6.0 (GraphPad Software, San Diego, Ca). All data shown are from at least two independent experiments conducted in duplicate or triplicate and are expressed as the mean ± SD or mean ± SEM, as specified in the figures’ legends. For comparison between two means, Student’s unpaired *t* test was used. For comparisons of the mean values among groups, a one-way or two-way ANOVA followed by Bonferroni post hoc or Dunnett test, as specified, were used. Statistical significance was set at *p *< 0.05.

## Results

### Phenotypic analyses of neurosphere-derived cells

The phenotype of neurosphere-forming cells was routinely evaluated by flow cytometric analysis using antibodies directed against the stemness markers β-tubulin III, nestin, and SOX-2, as well as the lineage marker for glial cells, GFAP. These markers were also analyzed in relative adherent cells to highlight the differences with respect to neurosphere-forming cells (Table 1). In accordance with previous reports [8, 63, 64], the percentages of positive cells for β-tubulin III, SOX-2 and nestin were higher than 80%, together with high MFI values in U87MG neurospheres. The percentage of positive cells in U87MG adherent cells never exceeded 55% for β-tubulin III and SOX-2 and the MFI values were approximately four-five times lower than the relative neurospheres. The percentage of positive adherent cells for nestin ranged between 85 and 98% while the MFI value was half that detected in relative neurospheres. The percentages of T98G positive cells for β-tubulin III, SOX-2 and nestin were not different between neurosphere-forming and adherent cells; however, as illustrated in Table 1, the MFI values in neurospheres were clearly higher when compared to adherent cells. The marker of glial differentiation GFAP, even showing a comparable percentage of positive cells among adherent and neurosphere-forming U87MG and T98G cells, appeared strongly down-modulated on neurospheres, as indicated by MFI level and as expected.

**Table 1:** Immunophenotypic characterization of GBM adherent cells and relative neurosphere

β-tubulin	Sox-2	Nestin	GFAP
U87MG adherent cells			
% positive cells			
51.30 ± 4.21	50.57 ± 3.02	92.41 ± 6.84	69.91 ± 2.38
MFI			
45.21 ± 3.78	36.57 ± 2.47	249.38 ± 15.86	139.14 ± 11.70
U87MG neurospheres			
% positive cells			
89.47 ± 7.23	95.41 ± 8.21	96.31 ± 4.39	59.71 ± 3.40
MFI			
210.60 ± 10.30	129.77 ± 10.82	511.61 ± 34.90	60.69 ± 5.11
T98G adherent cells			
% positive cells			
80.52 ± 4.34	86.84 ± 6.40	89.40 ± 6.96	52.96 ± 4.01
MFI			
117.51 ± 9.87	44.58 ± 36.84	99.58 ± 7.43	58.88 ± 1.99
T98G neurospheres			
% positive cells			
89.88 ± 5.73	97.63 ± 7.70	96.64 ± 4.80	4.11 ± 0.98
MFI			
189.40 ± 13.85	90.03 ± 8.02	411.38 ± 36.11	28.00 ± 1.45

Percentage (%) and value of Median Fluorescence Intensity (MFI) of β-tubulin III, SOX-2, nestin and GFAP in U87MG and T98G maintained in standard culture conditions (adherent cells) and in DMEM/F12 medium serum free with EGF, b-FGF and B27 supplement for neurosphere generation. Data are from one representative out of two independent experiments in duplicates ± SD

### COX-2 expression and activity in GBM cell lines

**Fig. 2:**
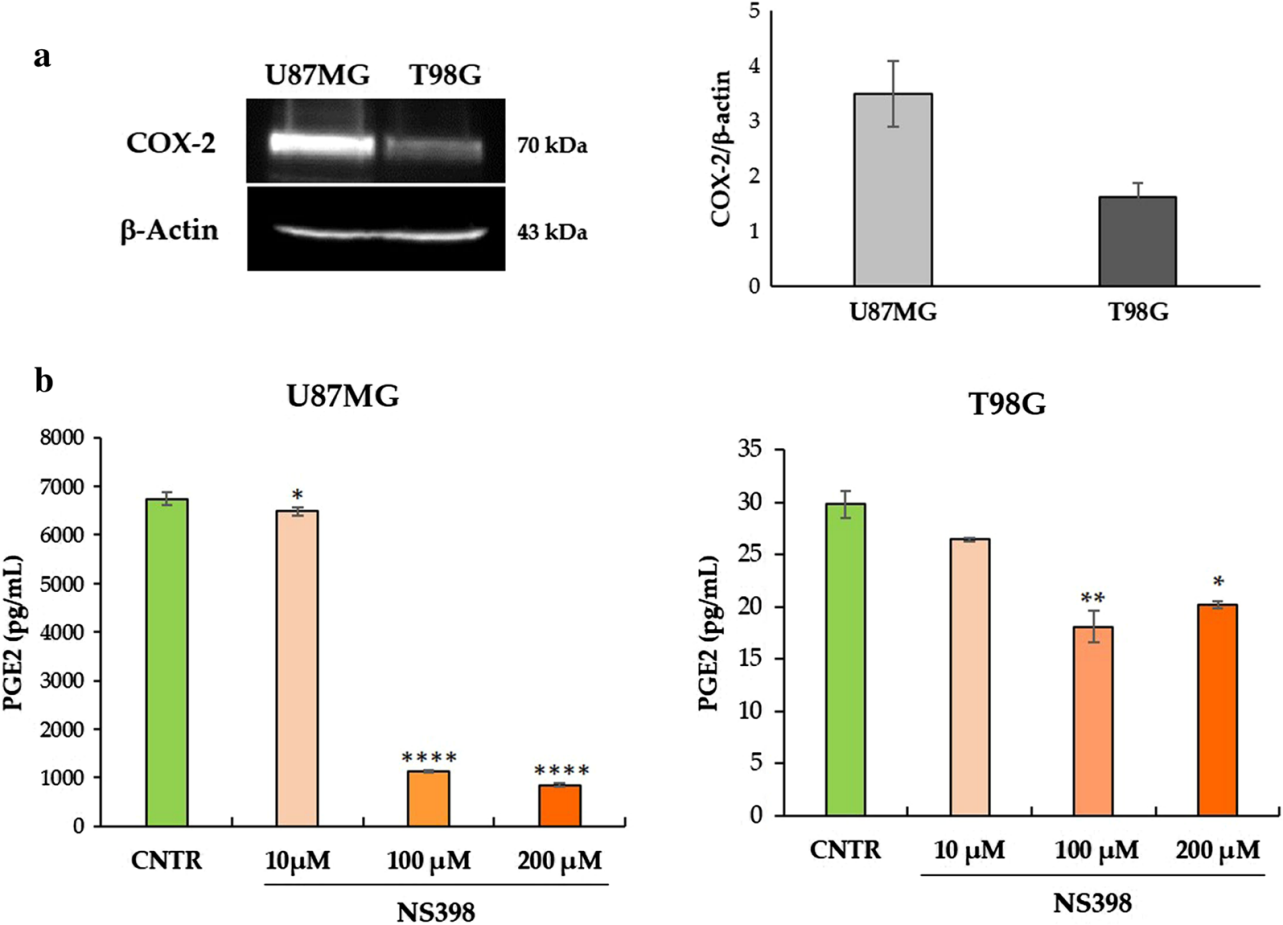
PGE2 levels released by U87MG- and T98G-derived neurospheres. **a** A representative image of western blot of COX-2 and β-actin proteins in control (CNTR) U87MG and T98G neurospheres. The results of the densitometric analysis of bands expressed as ratio *vs* β-actin band intensity are showed. Data are representative of three independent experiments, and mean values ± SEM are shown. **b** PGE2 levels were detected in supernatants from neurospheres after 48 h culture in the presence of NS398 at indicated concentrations. CNTR: control, drug vehicle alone. The results, relative to one representative out of two experiments performed in triplicate, are expressed as mean ± SD. For comparative analysis of groups of data, one-way ANOVA followed by Dunnett post hoc test was used (*p < 0.05, **p < 0.01, ****p < 0.0001 *vs* CNTR)

It has been previously reported the COX-2 expression in several GBM cell lines, including U87MG and T98G, and the basal levels of this protein varied greatly among them [65, 66]. To further confirm this evidence in our conditions, basal levels of COX-2 protein were evaluated in neurospheres generated from both U87MG and T98G cell lines. In Fig. 2a, a representative western blot is shown together with the relative densitometric analysis expressed as mean ± SEM from three independent experiments. To control the specificity of NS398 action, the modifications of PGE2 levels were measured following inhibitor addition at concentrations of 10, 100 and 200 μM, according to previous studies where similar levels of NS398 have been used in vitro on several types of cancer cells, including GBM cell lines [14, 34, 67]. The treatment with NS398 led to a significant decrease of PGE2 in the extracellular medium of the U87MG-derived neurospheres. The effect was dose-dependent being much more intense at high concentrations of NS398 (*p *< 0.0001 *vs* control) compared to 10 µM (*p *< 0.05 *vs* control) (Fig. 2b). In neurospheres derived from T98G cells, which basically released greatly lower amounts of PGE2, the addition of COX-2 inhibitor led to a significantly reduced PGE2 release at 100 µM (*p *< 0.01 *vs* control) and at 200 µM (*p *< 0.05 *vs* control) (Fig. 2b). The different expression of COX-2 detected in the U87MG- and T98G-neurospheres could explain the different basal PGE2 levels as well as the different effectiveness of the NS398 inhibitor. The control group was treated with drug vehicle DMSO (0.6% v/v) and indicated for all experiments as control.

### NS398 influenced neurospheres’ growth and morphology without affecting viability

**Fig. 3:**
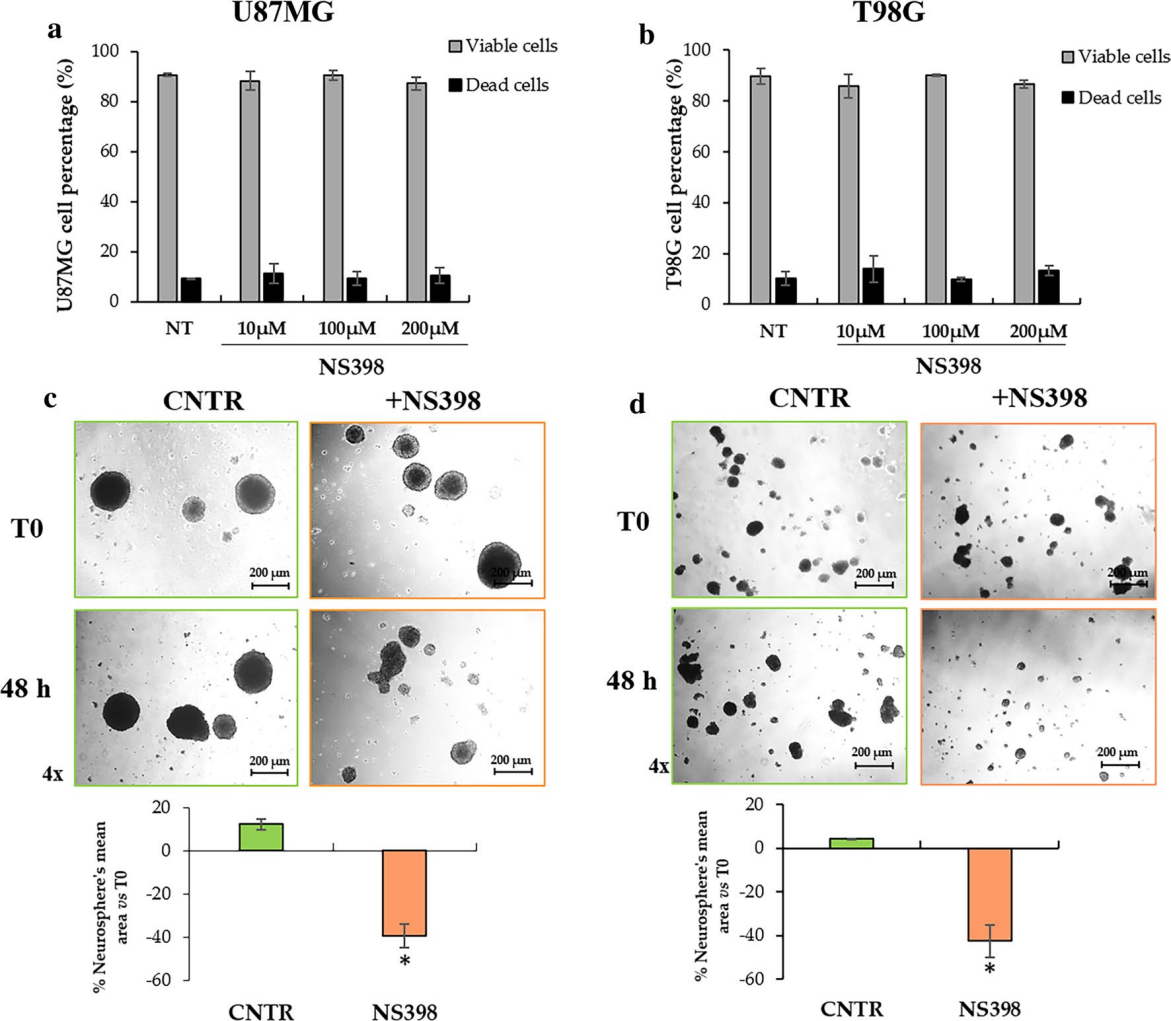
NS398 strongly affects GBM-derived neurospheres’ growth, without influencing cell viability. **a**, **b** Neurosphere-derived cell viability evaluated by flow cytometric analysis with PI staining. The percent viable and dead cells are reported in the histograms. **c** Representative phase-contrast images (4× magnification) of U87MG neurospheres in the absence (DMSO-treated, CNTR) or presence of COX-2 activity inhibitor NS398 (100 μM for 48 h). The quantitative analysis of neurospheres’ mean area was expressed as percentage *vs* relative T0. **d** Representative phase-contrast images (4× magnification) of T98G neurospheres in the absence (CNTR) or presence of COX-2 activity inhibitor NS398 (100 μM for 48 h). The quantitative analysis of neurospheres’ mean area was expressed as percentage *vs* relative T0. The results are representative of two independent experiments and are expressed as mean values of duplicates ± SEM. For comparison between two means, Student’s unpaired t-test was used (*p < 0.05)

Firstly, we examined if the NS398 could affect the glioma stem cell (GSC) viability and we observed that the exposure to NS398 inhibitor at the concentrations of 10, 100, and 200 µM did not significantly influence the percentage of viable U87MG- and T98G-derived neurospheres (Fig. 3a, b, respectively). DMSO, used to dissolve NS398 at 0.6% (v/v) final concentration, did not have any effects on its own. Based on these results and accordingly to the suggestions of Joki et al. [14], the concentration of NS398 100 µM was chosen for the subsequent experiments.

NS398 addition was able to significantly affect either neurospheres’ growth or morphology. Representative images acquired with a phase-contrast microscope (4× magnification) of U87MG- and T98G-derived neurospheres, incubated for 48 h with or without NS398 (100 μM) are shown in Fig. 3c, d, respectively. As evident, NS398-treated neurospheres from both GBM cell lines resulted smaller than control ones, the average area of control-U87MG spheres *vs* relative T0 was increasing by 12.22% ± 1.82 (mean value ± SEM); conversely, NS398-treated U87MG neurospheres’ area was reduced by 39.31% ± 3.88 (mean value ± SEM) *vs* relative T0 (*p *< 0.05) (Fig. 3c). Similarly, in T98G cultures, where the spheres at T0 appeared smaller than those of the U87MG, a significant reduction of relative mean area after the treatment with COX-2 inhibitor was also detected (-42.51% ± 7.45; * *p *< 0.05) (Fig. 3d). The effect of the drug could be detected in both cellular lines up to 72 h with a reduction of ~ 43% and ~ 47% on U87MG and T98G cells, respectively. At a longer incubation (range 96-144 h), after subculturing, with fresh medium and without inhibitor, the neurospheres’ mean area did not appear significantly different between control and treatment with NS398. This result could be due to the half-life of the drug. Indeed, as previously reported on in vitro models [68], NS398 should be replenished every 3 days. In this regard, experiments with long-term cultures of neurospheres involving the periodic addition of NS398 to cells will be required.

**Fig. 4:**
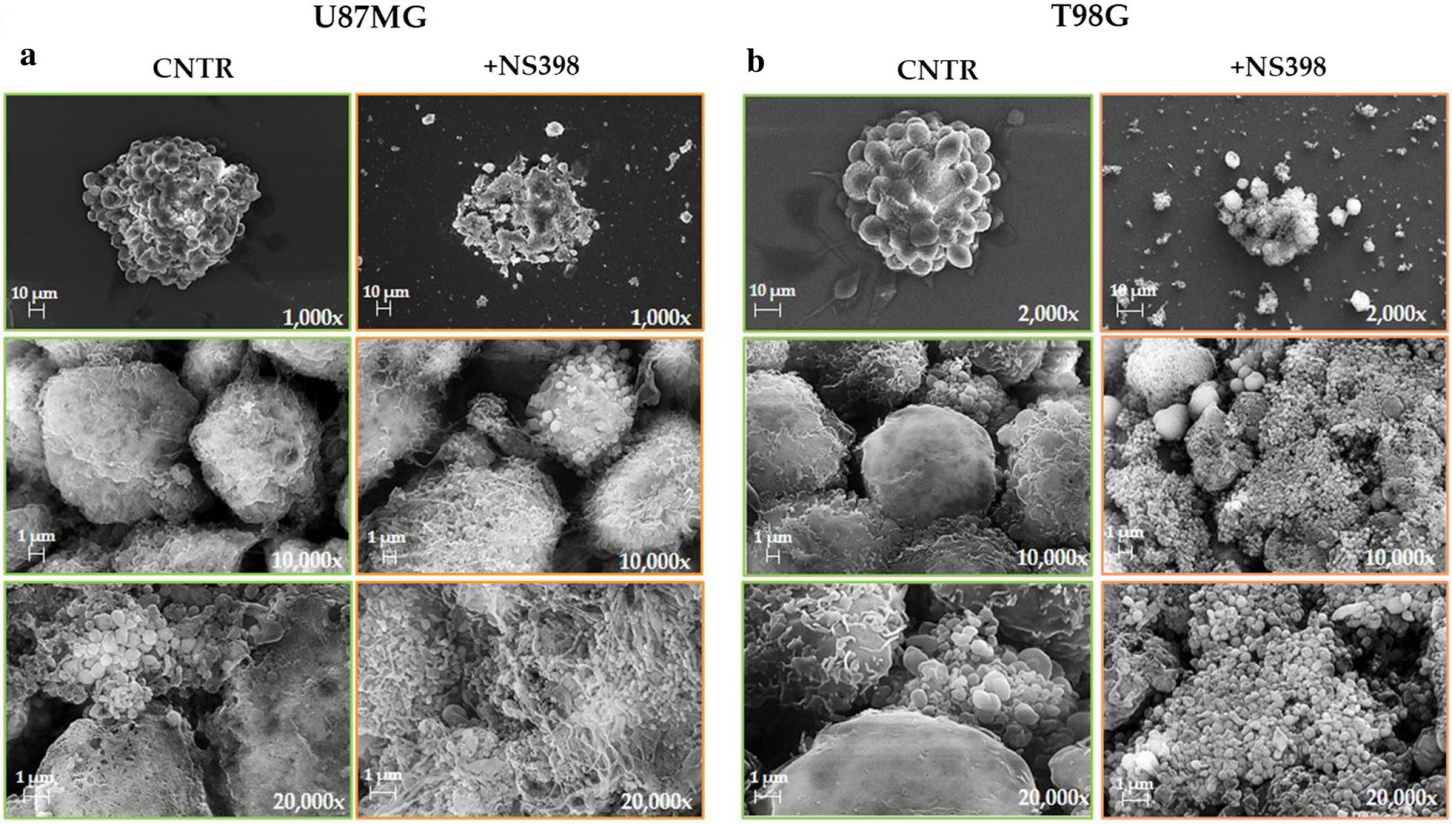
NS398 affects the neurospheres’ morphology. Representative SEM images of U87MG- and T98G-derived neurospheres (**a**, **b**, respectively) after 48 h treatment with vehicle alone (CNTR) or 100 μM NS398

To further assess the effects of NS398 on neurospheres’ morphology, images of U87MG and T98G neurospheres treated or not with NS398 were acquired at scanning electron microscopy (SEM) at several magnifications. Figure 4 shows SEM representative micrographs of U87MG and T98G neurospheres cultured for 48 h with or without NS398 (100 µM). The results obtained by SEM were overall overlapping with those from light microscopy: images at low magnification clearly highlighted a reduction of neurospheres’ size in treated cultures when compared to untreated ones. In addition, all NS398-treated neurospheres showed an alteration of the morphological features, being smaller, not well structured, with a clear cell disaggregation and extremely irregular outline. The membranes of treated neurospheres appeared much rougher compared to untreated spheres and were characterized by the presence of abundant vesicle-like membrane protrusions (Fig. 4a, b).

### NS398 induced autophagy in neurosphere cultures

**Fig. 5:**
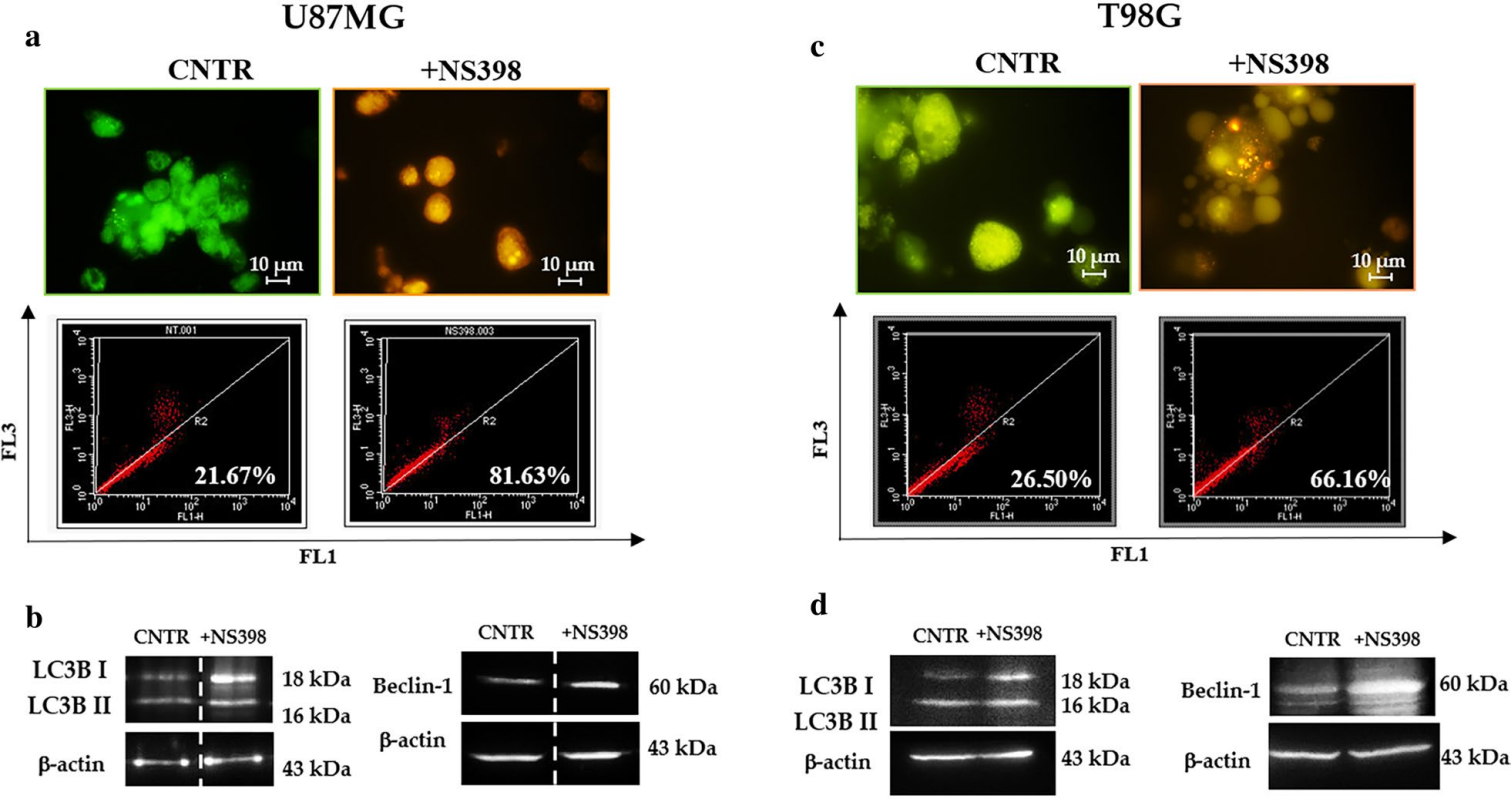
NS398 induces autophagy in GBM neurospheres. **a**, **c** Representative images of U87MG- and T98G-derived neurospheres treated with or without 100 µM NS398 for 48 h and stained with AO. The control cells were treated with DMSO alone (CNTR). Flow cytometric detection of red and green fluorescence of AVOs in U87MG- and T98G-derived neurospheres treated or not (CNTR) with 100 µM NS398 (48 h) is shown. **b**, **d** Representative western blots of LC3B I/II and Beclin-1 in U87MG- and T98G-neurospheres. The results are representative of three independent experiments

In order to verify whether NS398 could induce the autophagy pathway in GBM stem cells, fluorescence staining with acridine orange (AO) of the disaggregated neurospheres was assessed to visualize acidic compartments. In untreated cells, the cytoplasm and nuclei fluoresced bright green, while the treatment for 48 h with NS398 at 100 μM, caused a marked induction of AVOs in the cell cytoplasm as clearly shown by the bright orange/red acidic compartments (Fig. 5a, c). Furthermore, the quantitative FACS analysis of AVOs confirmed a significant increase in the fluorescence intensity as compared to control cells in both GBM cell lines (Fig. 5a, c). To confirm whether NS398 induced autophagic death, the classical autophagy-related proteins were evaluated. According to previous reports [69, 70], both LC3B I and LC3B II resulted upregulated in the cells treated with NS398. In particular, a consensus has emerged whereby overall levels of LC3B-II should be normalized to a loading control, such as β-actin [69]. In line with the molecular changes associated to autophagic process, Beclin-1 levels were also upregulated in NS398-treated neurospheres in comparison to control cells (Fig. 5b, d).

### Effect of EV released by U87MG- and T98G-derived neurospheres following NS398 treatment on adherent cell lines

**Fig. 6:**
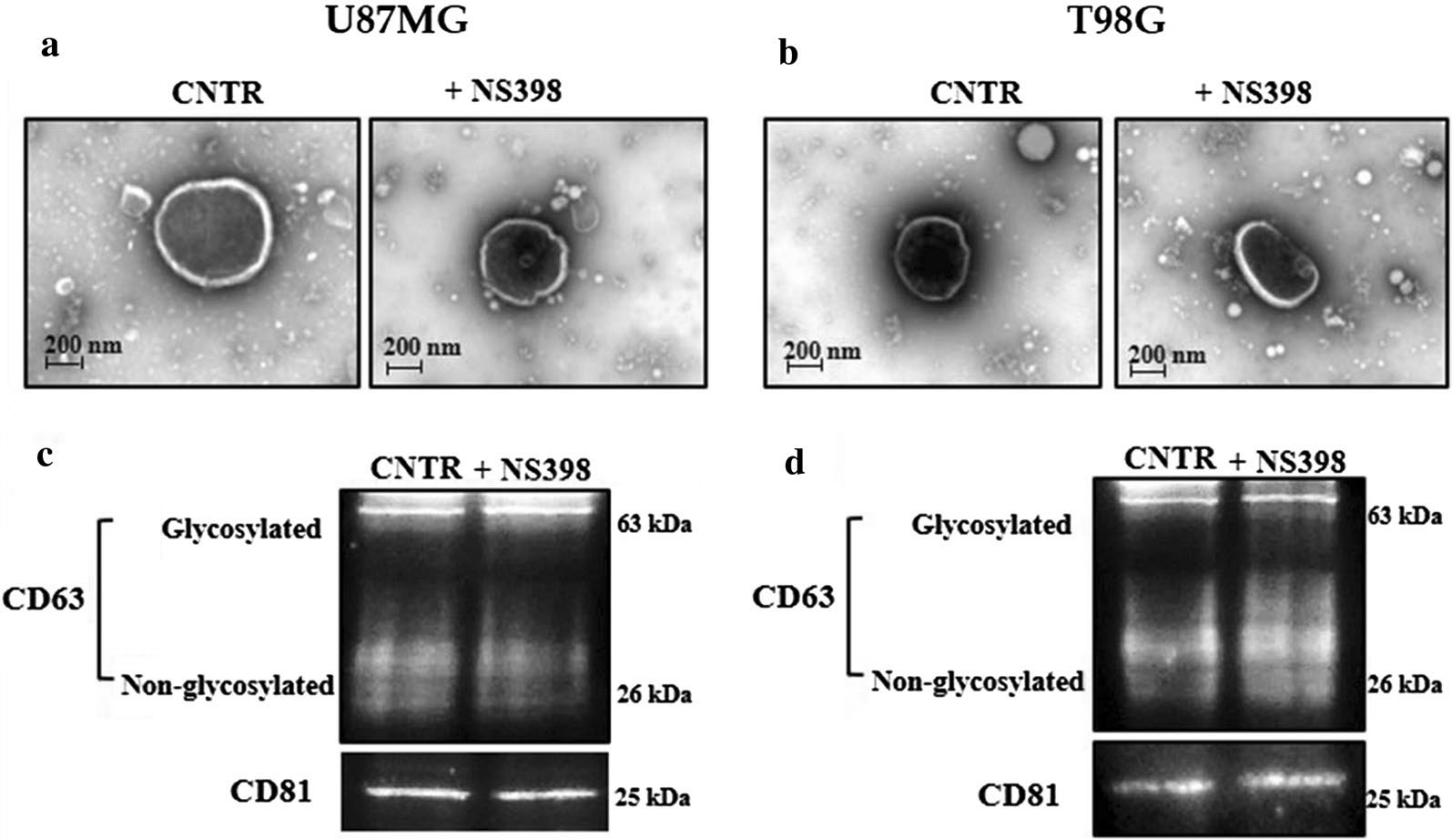
Characterization of isolated EV released by U87MG- and T98G-neurospheres’ cultures. Representative TEM images of EV from **a** U87MG- and **b** T98G-derived neurospheres cultured in the absence (CNTR) or presence of NS398 (100 µM) (50,000× magnification, scale bar 200 nm). Expression of EV specific markers CD63 and CD81 in EV collected from **c** U87MG- and **d** T98G-derived neurospheres treated with NS398 (100 µM, 48 h) or not (CNTR) was detected by Western

The ability of NS398 to modulate EV shedding from U87MG and T98G neurospheres was evaluated. After exposure of neurospheres for 48 h to NS398 at 100 µM, EV fraction, purified from supernatants and characterized by transmission electron microscopy (TEM), showed an unbroken bilayer membrane with a typical rounded morphology similar to EV released by DMSO-treated neurospheres (CNTR) (Fig. 6a, b). The tetraspanins CD63 and CD81 proteins, specific EV markers [71, 72], further confirmed the nature of isolated EV derived from NS398-treated or untreated neurospheres (Fig. 6c, d).

**Fig. 7:**
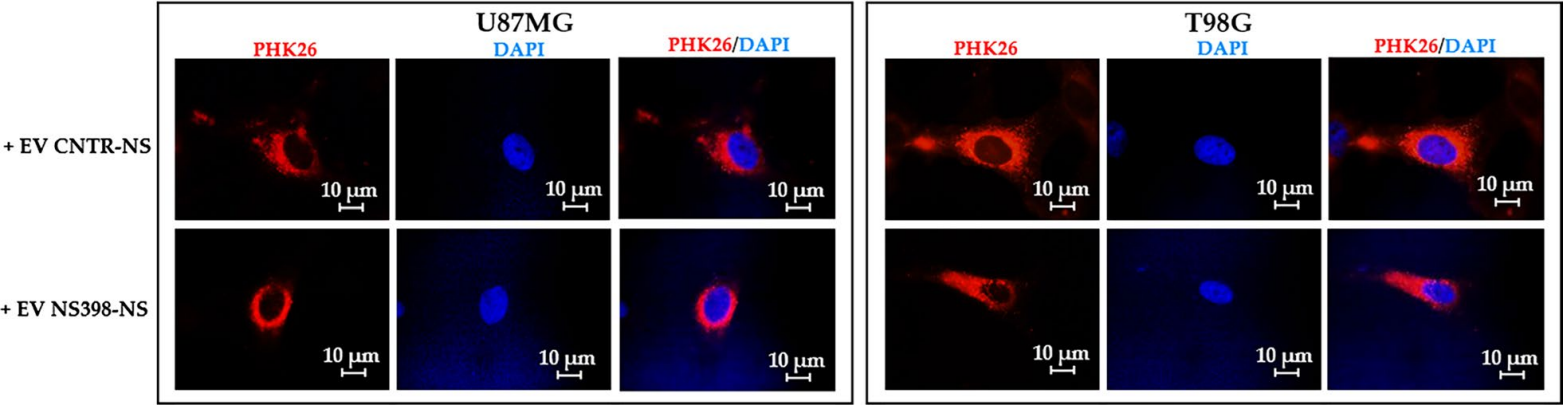
Uptake of EV derived from control (CNTR) and NS398-treated neurospheres on adherent U87MG and T98G. The EV uptake by recipient cells was evaluated by PKH67 dye (red) or Dapi dye (blue) to label EV and cell nucleus, respectively. Merged images for PHK67 and DAPI are also shown. Adherent U87MG and T98G cells were cultured with EV obtained from relative neurosphere cultures untreated (+EV CNTR-NS) or treated with NS398 (+EV NS398-NS). Representative images from two independent experiments are shown

The microscopic analysis showed that PKH26-labeled EV were effectively internalized by adherent GBM cell lines after 18 h of culture. The treatment of neurospheres with NS398 did not influence the EV uptake in adherent recipient GBM U87MG and T98G cells, resulting similar in all samples (Fig. 7). Adherent GBM cells were exposed to EV released by neurospheres previously cultured with NS398 at 100 µM for 48 h or with DMSO-drug vehicle (CNTR).

**Fig. 8:**
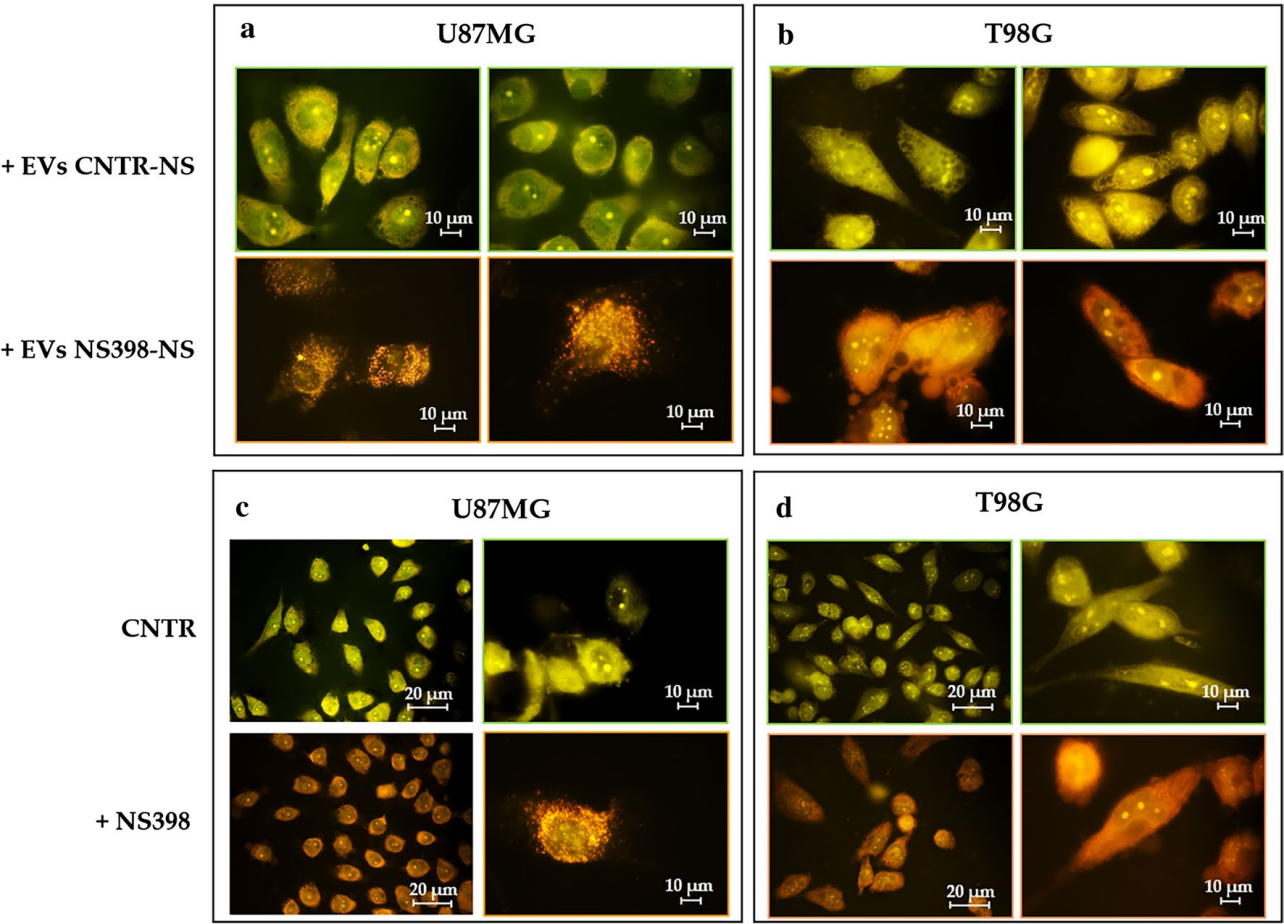
Indirect and direct induction of autophagy by NS398. **a** U87MG- and **b** T98G-treated with EV obtained from control (+EV CNTR-NS) and NS398-treated neurospheres (+EV NS398-NS). **c** U87MG- and **d** T98G-directly exposed to NS398. Two images for each condition are shown and are representative of two independent experiments. CNTR = DMSO-treated cells

EV secreted by NS398-treated neurospheres were able to induce high levels of AVO’s accumulation highlighted by a diffuse orange/red fluorescence in both recipient adherent cell lines (100%) (Fig. 8a, b). Conversely, the addition of EV released by DMSO-treated neurospheres had no effect in terms of AVO formation (0%). In Fig. 8c, d, the results of experiments aimed to evaluate the effects on autophagy of direct treatment with COX-2 inhibitor are shown. It is evident that the direct addition of NS398 led to an AVO level increase in both GBM cell lines which was similar to that observed after addition of EV released by NS398-treated neurospheres (100% *vs* 0% control).

### NS398 inhibitor impaired migration of adherent GBM cell lines

**Fig. 9:**
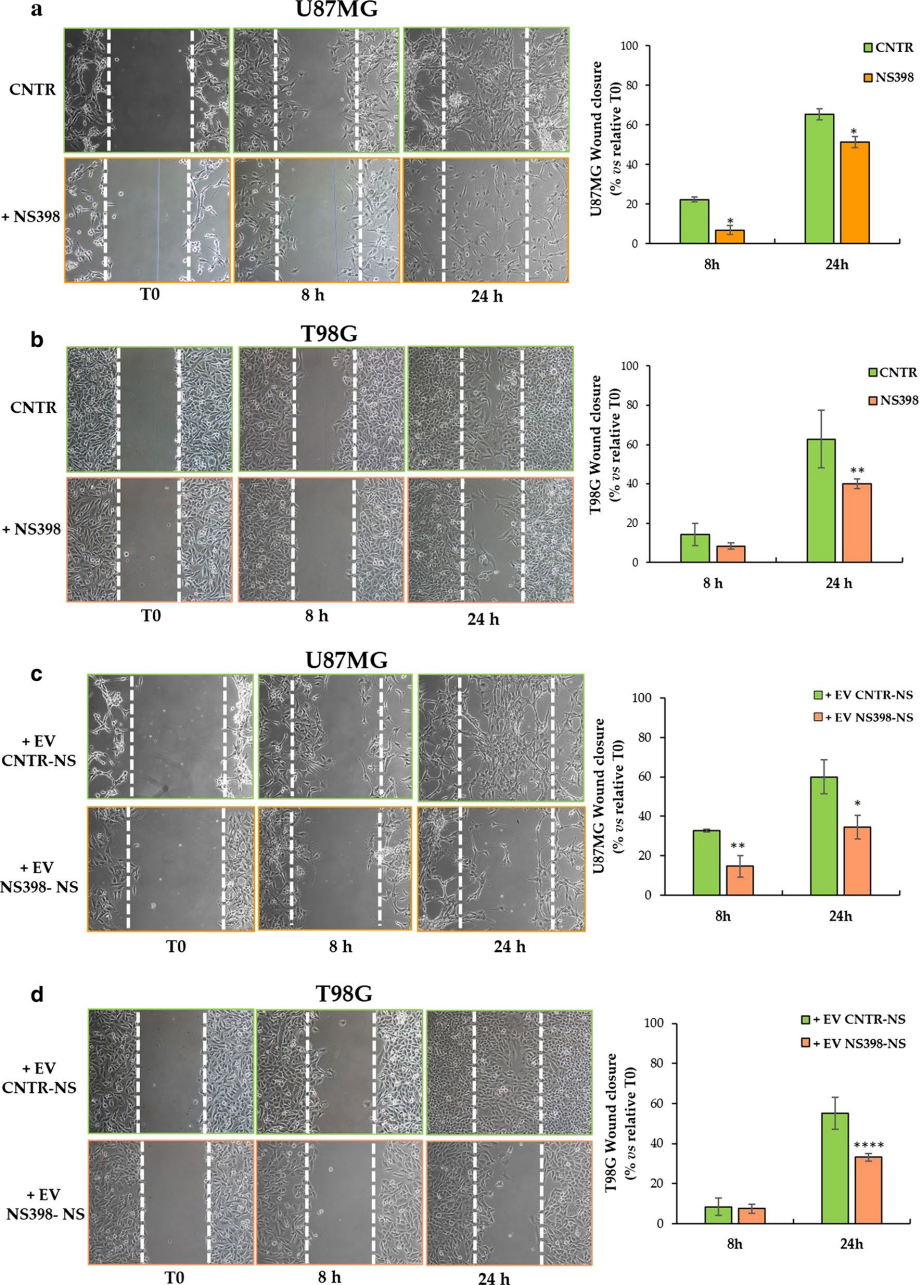
NS398 inhibited wound healing ability of GBM cell lines. Representative phase-contrast micrographs and quantification of scratch assays performed in **a** U87MG and **b** T98G GBM cell lines treated with NS398 or with drug vehicle DMSO (CNTR), immediately after the scratch at the initial time (T0) and after 8 h and 24 h. Representative phase-contrast micrographs and quantification of scratch assays performed in (**c**) U87MG and **d** T98G GBM cell lines after incubation with EV derived from control (CNTR) neurospheres (+EV CNTR-NS) or NS398-treated neurospheres (+EV NS398-NS). Original magnification 10x. The results, expressed as  % wound closure *vs* relative T0 (mean ± SD), are representative of two independent experiments in duplicate. For comparative analysis of groups of data, two-way ANOVA followed by Bonferroni post hoc test was used (*p < 0.05, **p < 0.01, ****p < 0.0001)

The capability of COX-2 inhibitor to influence tumor cell migration was evaluated using an in vitro artificial wound model. Figure 9 shows representative microscopy images at T0, 8 h, and 24 h, and the relative quantitative analysis of wound closure rate. The scratched monolayers of DMSO-treated cells (CNTR) were closed at ~ 25–28 h. Treatment with NS398 significantly delayed the U87MG monostrate repair both at 8 h (22.40 ± 1.08, CNTR *vs* 6.94 ± 2.4, NS398; *p *< 0.05) and 24 h (65.44 ± 2.89, CNTR *vs* 51.41 ± 2.82, NS398; *p *< 0.05) (Fig. 9a). Similarly, NS398 treatment negatively influenced the wound closure rate of T98G cells being the effects statistically relevant just at 24 h (62.79 ± 14.53, CNTR *vs* 39.95 ± 2.48, NS398; *p *< 0.01) (Fig. 9b). Under these conditions, while cell viability was not affected by NS398 treatment, the proliferation rate of both cell lines decreased compared to control cells, although to a different extent. The cell growth of U87MG cells was most affected by NS398, with a reduction rate of 10–12% and 25–30% at 8 and 24 h, respectively, while the number of T98G cells treated with NS398 was 5–7% and 10–15% lower than control cultures at 8 and 24 h, respectively. GBM cell lines show differences also in migration rate. Moreover, the scratch assay test showed that T98G grow with a much higher density than U87MG raising the question if cell density can affect the migration rate. However, with this model, we calculated the surface of the wounded area at 8 or 24 h compared to the initial area (T0), as measured immediately after scratch and considered 100%, regardless of density of the initial monolayer. The ability of the EV secreted from control (CNTR) and NS398-treated neurospheres to influence GBM adherent cell migration was also evaluated. The addition of EV from NS398-treated neurospheres significantly delayed the re-epithelialization of both U87MG and T98G scratched monolayers respect to relative T0 (Fig. 9c, d). In U87MG monolayers the percentage of wound closure rate at 8 h was 14.69 ± 0.41 with EV derived from NS398-treated neurospheres (+EV NS398-NS) *vs* 32.82 ± 1.58 of EV derived from untreated neurospheres (+EV CNTR-NS) showing a significant difference (*p *< 0.01). Moreover, the percentages of re-epithelialization at 24 h was 34.54 ± 1.34 with EV NS398-NS *vs* 60.03 ± 8.58 of EV CNTR-NS (*p *< 0.05) (Fig. 9c). The inhibitory effect of EV from NS398-treated T98G neurospheres resulted significant at 24 h after scratch (*p *< 0.0001) (Fig. 9d). These results suggested that both GBM cell lines exposed to EV purified from NS398-treated neurospheres media for 48 h showed a delay in re-epithelization respect to cells exposed to EV from DMSO-treated neurospheres (CNTR).

## Discussion

Previous studies revealed that COX-2 is expressed in most human malignant glioma cell lines cultured both in adherent condition and in a specific medium that favors stem cell growth [73–75]. COX2 expression was found highly associated with increased cancer stem cell population in multiple types of cancer, including glioma [30]. The glioma-promoting effects of COX-2 induction are largely mediated by its product PGE2 [76]. Similar to COX-2, elevated levels of PGE2 favor tumor initiation and progression and are also involved in the development of therapeutic resistance partially through affecting tumor microenvironment. Since PGE2 mainly functions through binding and activating prostaglandin receptors (EPs), together with COX-2 and EP, PGE2 forms a functional COX2/PGE2/EP axis with important biological and pathological functions [24]. In order to counteract the inflammatory process in GBM, the use of selective COX-2 inhibitor drugs is currently evaluating being considered as an adjunct treatment, also owing to their ability to increase GBM sensitiveness to traditional chemotherapy and radiotherapy [12].

To further explore the effects of COX-2 pharmacological inhibition in GBM, we investigated the effect of NS398, a highly selective COX-2 inhibitor, on human U87MG and T98G cell lines. NS398, even if not yet approved by the Food and Drug Administration (FDA) for clinical use, has been largely studied in vitro and in vivo on several types of cancers [77].

In our experimental conditions, neurospheres derived from both cell lines resulted highly influenced by treatment with NS398, showing morphological changes, a reduced growth rate, and a relevant level of autophagy. In addition, COX-2 inhibitor exposure could be associated with a surprising functional modification of the EV released by neurospheres. In fact, EV derived from neurospheres, pre-treated with NS398, strongly reduced the migration ability and caused a dramatic level of autophagy in the adherent cells of U87MG and T98G, thus causing effects quite similar to those observed following direct addition of the inhibitor. These results could be attributed to the potential effect of the inhibitor on the cargo of EV released by neurospheres or the possibility that NS398 added to neurospheres was trapped in the EV themselves which in turn could act as drug delivery vehicle [78]. In this context, further research will need to assess the influence of NS398 on cargo contents through next generation sequencing. Of note, our findings suggest that both GBM cell lines were equally influenced by NS398, despite the intrinsic diversity and individual genetic features, such as the TP53 gene status, MGMT activity and base excision repair (BER) or BRCA1 pathways, which in turn are associated to a different sensitivity or resistance to TMZ [55, 58, 59]. In our hands, even if both expression and enzymatic activity of COX-2 were significantly higher in U87MG than T98G, the COX-2/PGE2 inhibitor led to similar effects in terms of cell growth and viability, autophagy, cell migration. Of interest, Kuipers et al. (2007) showed evidence that both the cell growth and radiation-resistance either of COX-2 positive (U87) or negative (D384, U251) GBM cell lines were effectively inhibited by COX-2 inhibitors, including NS398 at 200 μM. These results led the authors to conclude that the effects of non-steroidal anti-inflammatory drugs (NSAIDs) could be independent on enzyme expression and probably caused by interaction with other targets than COX-2 [34]. In 2013, Gurpinar et al. published a detailed description of the major COX independent mechanisms of NSAIDs and COX-2 inhibitors, including inhibition of cyclic guanosine monophosphate phosphodiesterases (cGMP PDEs), Wingless-related integration site (Wnt) signaling, peroxisome proliferator-activated receptor δ (PPARδ) activity, phosphatidylinositol-3-kinase (PI3K)/3-phosphoinositide-dependent kinase-1 (PDK-1)/Protein Kinase B (Akt) pathway [79]. In line with these findings, our results show that U87MG and T98G are similarly sensitive to NS398 treatment, irrespective of the relevant difference in COX-2 expression and activity. So, further experiments with negative GBM COX-2 lines will be necessary to check whether the mechanism behind the observed effects of NS398 could be COX-2 independent. It should be of interest to also evaluate the influence of NS398 on healthy human brain astrocytes. In addition, given the functional implications of COX-1 in cancer the possible involvement of COX-1 should be evaluated in our experimental conditions [80].

Wu et al. [22] have recently reported that COX-2 inhibition with indomethacin, celecoxib, or siRNA against COX-2, enhanced the cytotoxic effect of TMZ on GSC isolated from primary GBM. Based on these findings, the comparable sensitivity observed in the two cell lines to NS398, despite the different sensitivity to TMZ, could be of even greater interest if substantial evidence will be provided about the ability of inhibition of COX-2/PGE2 system to convert a TMZ-resistant glioma cell to TMZ-sensitive.

Finally, even if in the present work we did not use normal astrocytes, several in vivo and in vitro reports showed that NS398 doesn’t exert cytotoxicity on normal astrocytes and neurons, including human brain astrocytes and neurons. Indeed, studies with this COX-2 inhibitor mostly highlight its protective effects in neuroinflammation-associated disease models or high toxicity conditions [81–86].

## Conclusions

Altogether, our results strongly support the role of the COX-2/PGE2 system in glioma and glioma stem cell biology. The COX-2 inhibitor influenced in a similar way both investigated GBM cell lines i.e., U87MG and T98G, despite their intrinsic diversity and individual genetic features, which in turn are associated to a different sensitivity or resistance to TMZ. Although further studies are needed to gain a complete picture of the mechanisms involved in the observed effects, overall, our data help to broaden the range of biological effects caused by NS398.

## Data Availability

The datasets generated and analyzed during the current study are available from the corresponding authors upon reasonable request.

## Abbreviations

GBM: Glioblastoma multiforme
GSCs: Glioma stem cells
COX-2: Cyclooxygenase-2
PGE2: Prostaglandin E2
NS398: *N*-[2-(cyclohexyloxy)-4-nitrophenyl]-methanesulphonamide
EV: Extracellular vesicles
NS: Neurospheres
AVO: Acidic vesicular organelles
AO: Acridine orange
